# Supplementary material for: Complexome profiling of the Chlamydomonas psb28 mutant reveals TEF5 as an early PSII assembly factor
Source: Plant Cell. 2025 Mar 17;37(6):koaf055. doi: 10.1093/plcell/koaf055 (PMC12164590; doi:10.1093/plcell/koaf055)
Supplement: koaf055_Supplementary_Data [file koaf055_supplementary_data.zip › psb28_interactive.zip!/plots/intensity/qualityPlots/peptides/_Cre01.g032750.t1.2.html]

### Cre01.g032750.t1.2

Trivial name:   
SeqCoverage [median]: 31.00  
Mass: 18.81 kDa  
  
 

| Sequence | MissedCleavage | Mass [Da] | wtIntensity | muIntensity | Score |
| --- | --- | --- | --- | --- | --- |
| ALAITLNR | 0 | 870.53 | 870.81 | 3124.24 | 81.30 |
| ASLNEIR | 0 | 801.43 | 705.93 | 4633.95 | 150.84 |
| IAEFQEEVQSLASK | 0 | 1577.79 | 717.47 | 6571.90 | 164.48 |
| IAEFQEEVQSLASKEMSR | 1 | 2081.01 | 1276.75 | 0.00 | 77.47 |
| QAGAAAGTGAVAGVAAGAGAGSQS | 0 | 1856.90 | 332.48 | 604.06 | 71.65 |
|  |  |  | 3903.4 | 14934.2 |  |
